# The New York Institute of Stomatology

**Published:** 1897-12

**Authors:** 

**Affiliations:** The New York Institute of Stomatology


### THE NEW YORK INSTITUTE OF STOMATOLOGY.

A REGULAR meeting of the Institute was held Tuesday evening, October 5, 1897, at the residence of Dr. Benjamin Lord, No. 34 West Twenty-eighth Street, New York City, the President, Dr. George S. Allan, in the chair.

The minutes of the previous meeting were read and approved.

*Dr. W. St. George Elliott.*—I would like to ask for some opinion in regard to a little difficulty I have had in the cataphoric destruction of pulps. I have had two cases where there was marked sensibility of the surface of the tooth and a great deal of pain on attempting to drill. While both were very sensitive, in neither case was there any pulp structure apparent. Yet the pain entirely subsided under cataphoric treatment and the canals were drilled out.

*Dr. J. Morgan Howe.*—Does Dr. Elliott mean that the pulp-canals were filled by secondary dentine?

*Dr. Elliott.*—Partly, but not completely. In one case there was a little hemorrhage at the extreme end; in the other case no organic matter at all, yet the extreme sensitiveness was there.

*Dr. S. C. G. Watkins.*—Was the canal large enough to insert a broach?

*Dr. Elliott.*—Yes, a very fine broach.

*Dr. C. F. Allan.*—If there were no pulp, and if the canals were not completely ossified, it is difficult to suggest or see what there could have been in the teeth to give pain.

*Dr. Elliott.*—My impression is that there was a pulp in both cases, but of exceedingly filamentous character and not readily recognized.

*Dr. C. F. Allan.*—I will speak of the obliteration of two pulps, one of a superior cuspid and the other of a central incisor, by entirely different means, under different circumstances, both accomplishing very excellent results. In the case of the central incisor, a blow had been received from a golf club, the tooth crown being splintered. It was in a number of pieces, seemingly held together solely by the central connection with the pulp,—splintered up on the labial and palatal face of the root, about a quarter of an inch. The patient came to me just at dusk, suffering a great deal, and the only thing I could think of doing at the time was to make a splint of German-silver plate, very thin, putting pieces smeared over with oxyphosphate on the front and back, simply tying them together to hold the splintered tooth in place until the next day, when I could do something better. That gave relief until his next appointment. When he came again, considerable pain was caused by the removal of the pieces of the crown. Having done that (the case being one in which cataphoresis could not be used, as the tooth could not be kept dry), I used trichloracetic acid, and in an hour had the pulp-chamber clean to the end of the root, and the patient said the pain was hardly worth mentioning. I used the actual crystals of the acid so as to have the most prompt action possible.

The other case was one of cataphoresis. That was a cuspid. The man was about sixty years of age, and the wear on the tooth had gone on more rapidly than the reparative process of the pulp, and the tooth was very sensitive, the tooth-structure between the outside and the pulp being quite thin. The pulp would have to be destroyed, because I wanted to "shoe" the tooth, as the term goes, with platinum and gold, making a surface as hard as possible. In that case I applied cataphoresis, and at the end of half an hour, giving it full time, as I thought, to make a complete penetration, I was enabled to take out the pulp without any trace of pain. This case was one well adapted for cataphoresis, and the result was what might have been expected.

*The President.*—We will now take up the subject of the evening. The Executive Committee some weeks ago sent to various gentlemen extracts from the conclusions and statements of Drs. Black and Williams that "liability of teeth to decay is determined only by environment and not by structure," and we are able to present this evening the replies that have been received. Every little while something happens in the profession that sets us all thinking. We have preconceived opinions and ideas on theory and practice, and they are more or less disordered by what we see and hear, sometimes of a very startling nature. After reading these views that Drs. Black and Williams have promulgated, and which have been so largely circulated in the magazines, most dentists put an interrogation mark to their ideas. In the hope of reaching the profession more generally, and of finding out to what extent these opinions have been accepted as true, or should become acknowledged as true in practice, our Executive Committee has taken this means of having the discussion brought before the profession.

We have with us Dr. James Truman, of Philadelphia, and I will now call upon him to tell us his ideas of the subject.

*Dr. Truman.*—When I received the invitation to be present with you I was somewhat at a loss to know how I was expected to treat the subject. I subsequently found that this meeting was to be a sort of symposium on Drs. Black and Williams, and I concluded that it would be best to write out my views, and I, therefore, present them for your consideration.

(For Dr. Truman's paper, see page 796.)

The secretary then read papers by Drs. George A. Maxfield, R. R. Andrews, B. Holly Smith, E. A. Bogue, S. B. Palmer, and Mr. Charles Tomes.

(For these several papers, see pages 781–814.)

A letter from Dr. W. D. Miller, of Berlin, was read, stating that as the writer had been prevented by ill health from studying thoroughly the published accounts of the recent work of Drs. Black and Williams, he felt that any expression of opinion by him would be unfair and without value.

*The President.*—We also have a short communication from Dr. W. H. Potter, of Boston, which the secretary will now read.

*Dr. W. H. Potter.*—I am of the opinion that in the matter of dental caries there are two factors to be considered:

1. The condition of tooth-tissue.
2. The environment of the tooth.

I feel confident from observations made in practice that some teeth offer a greater resistance to unfavorable environment than do others. I should, however, find it difficult to say which was the more important agent in producing caries, a bad environment or defective structure. I believe that as the lung-tissue, on account of the make-up of its substance and from its inherent vitality, can resist the attacks of tubercular bacteria, so the teeth by a superior chemical composition, joined with a vitality coming from the pulp, can resist the unfavorable effects of environment. It is not simply a chemical problem which we study but a chemico-vital one.

### DISCUSSION.

*Dr. C. S. Stockton.*—I have but little to say, as I am somewhat "at sea" on this subject. With eminent men on one side and equally eminent men on the other, their theories and practice seem to be directly opposite. It is very much like the charge of the justice of the peace to the jury,—"Gentlemen, if you believe the distinguished lawyer on the one side you will find a verdict for the plaintiff, and if you believe the equally distinguished lawyer on the other side, you will find a verdict for the defendant; but if you are like me, you won't believe either of them." Those of us who are laymen, so to speak, in this advanced scientific development regarding the destruction of teeth, do not know where we are. It seems strange that a man like Dr. Potter should make the statement he does. Of course we all know that the structure of the teeth and their environment are the two topics under discussion to-night, and that these two factors cause the destruction of teeth. Take what we call a soft tooth and put it in the surroundings that some hard teeth have, and that soft tooth will become a useful organ. Take what is termed a hard tooth with different surroundings and environments, and the tooth is lost. Take a child born into the world with the best circumstances and surroundings that can possibly exist, one who has been brought up with all the care that love can lavish upon it, place it, before its character is fully established, in the slums and places where it imbibes everything that is bad and evil, will it remain good? Will it adhere to the teachings and principles that it had when it was young? I think not. This is very largely so in regard to this matter of the decay of the teeth. Only this morning I saw a patient who takes the best care of her teeth; on the coronal surface of a cuspid, where it seems almost impossible for anything to lodge, was a cavity. What caused it? I do not know whether it was Professor Truman's theory of chemical action or Dr. Black's theory of microbes. These are times when the men who are leaders, and who have been for years making special study of this subject, should be able to tell those of us who do not know so much what it is that causes the destruction of teeth and how to remedy it.

*Dr. E. S. Niles.*—I came here rather to listen than to impart any ideas. We all have our own theories regarding decay, and could hardly practise our profession without them. During the past year I have had my mind directed upon new lines, which came to me through the use of nitrate of silver. We know practically that nitrate of silver is an antidote for decay; that if we apply it to a cavity where decay is progressing the decay is arrested. To me this is one of the most important facts that has come under my observation since the discovery of microbes and the presence of acids. I am not prepared to-night to give the results of some substitutes that I have been using to produce the same effects as nitrate of silver. My theory as to the practical action of nitrate of silver is this: nitrate of silver retains all the coagulating power that is produced with nitric acid. Metallic silver in itself is negative; in most of its salts it is somewhat antiseptic; but it is more active in the nitric acid preparation. The great hinderance in the use of nitrate of silver is its discoloration when exposed to the light, masking its chemical action after a time. Where decay was abundant the yellow precipitate produced by nitrate of silver, observed before it turns black, gave me the hint as to its chemical action, and it at once called to my mind some experiments in which I proved to my mind the presence of an important factor in decay. The nitrate I then used will not discolor the caries, and I have had some satisfactory practical tests. It is my intention to give it to the profession a little later on. To me the practical results of these chemical changes furnished the clue to dental caries. I will tell how to produce this yellow precipitate out of the mouth. It is an old recognized reaction in chemistry. Take a solution of phosphate of lime and add a solution of nitrate of silver, the yellow precipitate will soon follow. The nitrate I refer to will cause the same reaction. I am impressed that there is in decay a retrograde metamorphosis or change of tooth structure in the tooth similar to that in digestion, where many factors are at work. One investigator finds germs, another acids. Scientists tell us that there are certain germs which are essential to perfect digestion. This question of decay is a complex one, and it has been well studied on certain lines, some of which have been brought before us to-night; but like the man who hunted for the mouse,—he searched all around the barrel while the mouse was in the meal in the barrel. We know beyond a doubt that there is acid in dental caries, but I am not convinced that it is wholly lactic acid, or acetic acid, or germs, because there are not materials enough about the teeth to keep up a constant supply of acid to do the amount of work found in decay.

*Dr. J. Morgan Howe.*—In the presentation of views it seems to me not necessary that they be regarded as antagonistic in an objectionable sense. Nobody antagonizes facts. We are thankful to Dr. Black and to Dr. Williams for the facts that they have given us. The differences of opinion that have been brought out are differences of deduction from the facts, not only from those presented by Drs. Black and Williams, but from the other facts accumulated from the store of knowledge that has come down to us from ancient times, from our own experiences, and from the record of the reliable observation of others. If different conclusions must be entertained than those arrived at by scientific observers as the results of their laboratory experiments, it is no disparagement of those who give us the facts obtained by research. Dr. Miller concluded from his experimental production of decay out of the mouth that teeth vary very much in their resisting power, as shown by the different conditions presented by numerous pieces of teeth subjected to the same influence; and I think it would be a fair test of the liability of teeth to the inception of decay to subject a number of them to tests in a similar way. We are not bound to accept the conclusions of any one when the conditions can be tested, but we gladly accept every fact that can be added to our store; and the testing of the question, whether environment is the only factor in the liability of teeth to decay, would give us another fact instead of a deduction.

*The President.*—There is a most astonishing unanimity of opinion in the various papers we have listened to this evening concerning the conclusions of Drs. Black and Williams. It would appear that the profession does not accept them as correct. It is a little surprising that no one writer or speaker has approved of them. The time will not permit me to ask for further opinions and remarks, so I will ask Dr. Truman to close the discussion, as he is the only representative of those who have written papers for us on this subject who is present.

*Dr. Truman.*—I think I practically stated my side of the subject in the paper I presented. I cannot quite understand in one of the papers why the question should be raised as to why micro-organisms flourish in tooth-structure. Miller fully settled that matter long since, that the excreta of the micro-organisms, or lactic acid, was taken up by the lime of the teeth, thus enabling the micro-organisms to flourish. We all know that micro-organisms die sooner or later in their own toxin.

Dr. Williams positively states that micro-organisms are the cause of enamel destruction. This I have endeavored to prove has not been demonstrated, and I think I could show, had I a blackboard, that those organisms can be found in all dense-structured teeth, and not in the chalky teeth to any considerable extent. Twenty-five or thirty years ago I explained to my classes that in all probability these were a powerful factor in producing dental caries in dense teeth. Those pictures represented in the illustrations so beautifully by Williams are, to my view, simply agents in holding the acids of the mouth against the enamel. As I stated in my paper, no authority that I know of has ever demonstrated that they are acid-producers, and I therefore criticise Williams on the point that he has stated things that have not been scientifically demonstrated, and when he undertakes to assume that caries is produced by those organisms he has gone beyond the region of facts.

Dr. Davenport moved a vote of thanks to Dr. Truman and to the authors of the various papers.

Carried unanimously.

Adjourned.

S. E. Davenport, D.D.S., M.D.S.,
*Editor The New York Institute of Stomatology.*

